# Piezoresistive Load Sensing and Percolation Phenomena in Portland Cement Composite Modified with In-Situ Synthesized Carbon Nanofibers

**DOI:** 10.3390/nano9040594

**Published:** 2019-04-10

**Authors:** Thanyarat Buasiri, Karin Habermehl-Cwirzen, Lukasz Krzeminski, Andrzej Cwirzen

**Affiliations:** 1 Building Materials, Department of Civil, Environmental and Natural Resources Engineering, Luleå University of Technology, 97187 Luleå, Sweden; karin.habermehl-cwirzen@ltu.se (K.H.-C.); andrzej.cwirzen@ltu.se (A.C.); 2 The Institute of Engineering Materials and Biomaterials, Silesian University of Technology, 44-100 Gliwice, Poland; lukasz.krzeminski@polsl.pl

**Keywords:** Carbon nanofibers, CVD, percolation, piezoresistive response, compressive load

## Abstract

Carbon nanofibers (CNFs) were directly synthesized on Portland cement particles by chemical vapor deposition. The so-produced cements contained between 2.51–2.71 wt% of CNFs; depending on the production batch. Several mortar mixes containing between 0 and 10 wt% of the modified cement were produced and the electrical properties at various ages and the load sensing capabilities determined. The percolation threshold related to the electrical conductivity was detected and corresponded to the amount of the present CNFs, 0.271, 0.189, 0.135 and 0.108 wt%. The observed threshold depended on the degree of hydration of the Portland cement. The studied mortars showed a strong piezoresistive response to the applied compressive load reaching a 17% change of the electrical resistivity at an applied load of 3.5 MPa and 90% at 26 MPa. This initial study showed that the studied material is potentially suitable for future development of novel fully integrated monitoring systems for concrete structures.

## 1. Introduction

Carbon-based materials, especially carbon nanotubes (CNTs) and carbon nanofibers (CNFs), incorporated into Portland cement based matrixes have been studied over the last couple of years. The solidified composite materials showed considerable tensile strength, an increased modulus of elasticity as well as improved thermal and electrical conductivity [1,2,3,4]. The nanofibers were shown to bridge cracks and hindered their propagation [5,6,7]. Furthermore, the hydration processes were enhanced by the formation of additional nucleation sites. The amount of formed calcium silicate hydrate (C-S-H) was reported to be increased while the total porosity decreased [4,8]. The excellent electrical properties of these composites enabled the production of matrixes having load, stress, strain, and crack formation sensing capabilities through piezoresistive response [9]. However, to function properly these composites require the presence of a sufficient amount of the conductive material, evenly dispersed throughout the isolative matrix to create a continuous electrical path. In tension, the electrical resistivity increases due to the progressive breakage of the conduction paths by the formation of cracks. In compression, the resistivity decreases due to fiber push-in which increases the probability that adjacent fibers will gain contact though the tunneling effect [10,11]. Konsta-Gdoutos and Aza [12] produced cementitious matrixes incorporating CNFs showing piezoresistive response under cyclic compression load in the elastic regime. Also other cement-based composites with conductive additions, e.g. carbon black/cement composites showed sensitivity to compressive and tensile strain [13,14,15].

Composite systems comprising of isolative and conductive phases may conduct an electrical current due to two mechanisms: Percolation in a continuous conductive network and/or tunneling [16,17,18,19]. The tunneling phenomena occurs when the inter-particle distance of the adjacent conductive particles is small enough to enable an electron to tunnel the gap and to form a conductive path [20]. Adding a small amount of conductive material to an insulating matrix only changes the electrical conductivity of the composite slightly as the isolated conductive particles do not form conductive networks. The critical volume fraction of a conductive phase at which a composite transforms from an insulator to a conductor is called the “percolation threshold”. The percolation theory assumes that conductive materials including fillers and fiber can create a conductive network within an isolative matrix if their amount is sufficiently high to provide contact points between them. The conductivity σ can be estimated by using a simple power law based on the statistical percolation Equation (1):

(1) $$\sigma \;\alpha \;{\left(p-p_{c}\right)}^{t}$$

where: p is the probability of occupation of a site in a resistor network by a conducting element, $p_{c}$ is the critical probability for bond percolation and *t* is the critical exponent. The equation is valid only for $p-p_{c}<<1$ when the fluctuations extend over distances which are much larger than the size of the constituents [21]. Based on Equation (1) the conductivity σ can be calculated according to Equation (2):

(2) $$\sigma =\sigma_{0}{\left(\Phi -\Phi_{c}\right)}^{t}$$

where: $\Phi_{c}$ is the concentration of conductive fibers or fillers corresponding to the percolation threshold, $\sigma_{0}$ is the conductivity of the conductive material and *t* is the critical index of conductivity with a theoretical value between 1.5–1.75 [22].

Within the traditional percolation theory, Equation (2) is based on the assumption that the electrical conductivity is only depended on the conductivity of the filler, in this case of the CNF. However, the maximum conductivity depends also on several other factors related to CNFs but also to the embedding matrix. Studies on polymer-based matrixes showed that increasing the aspect ratio between length and diameter (L/D) of the CNTs produced higher ultimate conductivity values [23]. The electrical conductivity $\sigma_{com}$ proposed by Hu et al [23] can be calculated according to Equation (3):

(3) $$\sigma_{com}=\sigma_{CNT}\cdot 10^{0.85\left\{\log\left(L/D\right)-1\right\}}\cdot {\left\{\Phi -\Phi_{c}\right\}}^{t}$$

where: $\sigma_{CNT}$ is the electrical conductivity of the CNTs, L is the length of CNTs, D is the diameter of CNTs, Φ is the volume fraction of the CNTs, $\Phi_{c}$ is the percolation threshold and *t* is a critical exponent determined experimentally.

More curly CNTs incorporated into polymer matrixes resulted in a higher percolation threshold and a lower maximum electrical conductivity [23,24]. The biggest challenge while incorporating CNTs/CNFs into cementitious matrixes is to obtain their uniform dispersion [6,25,26,27]. Their hydrophobic nature results in a strong tendency to agglomerate. One of the developed solutions was a direct synthesis of CNTs/CNFs on untreated Portland cement particles. The used method is based on the application of a chemical vapor deposition (CVD) process [28,29,30,31]. Iron (III) oxide (Fe_2_O_3_), a main component in Portland cement acted as the main natural catalytic substrate while the other components silica oxide (SiO_2_), magnesium oxide (MgO) and aluminium oxide (Al_2_O_3_) supported the growth of carbon nanomaterials [32,33,34]. Acetylene was used as carbon source and the synthesis temperatures ranged between 400–700 °C. The amount and the morphology of the synthesized nanofibers strongly depended on the duration of the process. After the hydration process the solidified matrixes showed twice the compressive strength and 40 times higher electrical conductivity than the reference samples. High electrical conductivity could indicate also a significant piezoresistive response to e.g., stress and strain variations [28,29]. The initial study presented in this publication aimed to determine that response. Solidified matrixes containing various amounts of the nanomodified Portland cement with synthesized in-situ CNFs were subjected to various compression loads and tested for the corresponding piezoresistive response. The long-term goal of this research is to develop a novel monitoring system, which would be fully integrated, or part of a concrete structure.

## 2. Experimental Details 

### 2.1. Synthesis and Characterization of SmartCem

The nanomodified cement (SmartCem) was produced using a CVD reactor (produced by the CVD Equipment Corporation) located in the laboratory of the Silesian University of Technology in Poland. Two types of SmartCem were produced using different process parameters (Table 1).

**Figure 1:**
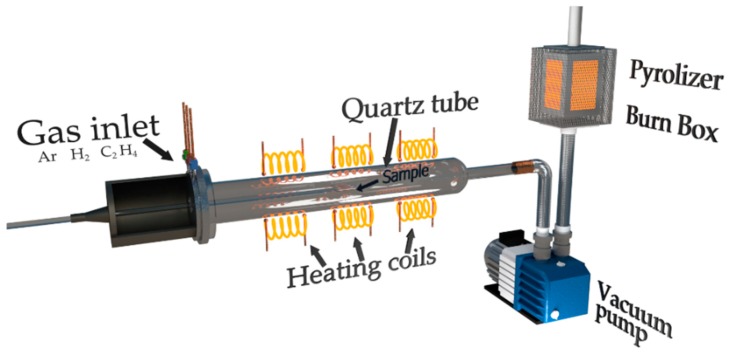
Schematic diagram of a Chemical Vapor Deposition (CVD) process for carbon nanofiber and SmartCem synthesis.

Ethylene (99.999%) was used as the precursor, hydrogen (99.99999%) as the reducer and argon as the transporting media. A total of 10 g of an ordinary Portland cement (CEM I 42.5) was used as a substrate for the synthesis of the carbon phase. The cement powder was placed in four parallel-arranged holders made of quartz having a length of 30 mm and a diameter of 8 mm. The holders were arranged according to the gas flow direction to enhance the reaction efficiency and to remove impurities (Figure 1).

The samples were placed in the quartz tube of CVD reactor having a diameter of 70 mm and a length of 50 cm. The samples were degassed by keeping them at a low pressure (0.001 mbar) at 90 °C for 60 min. In the following step, the temperature was raised to 110 °C and was kept at that level for 20 min to remove gaseous pollutants and moisture. In the next stage, the samples were heated to 740 °C at rate of 5 °C/min. This happened in argon atmosphere at a pressure of 1010 mbar. After stabilizing the temperature, the samples were subjected for 15 min to the reduction reaction in an atmosphere of a mixture of hydrogen and argon with flow rate of 500 sccm and 200 sccm, respectively. After the reduction of the catalyst surface, the reactor temperature was stabilized to 750 °C under 1 SLM argon flow, followed by releasing a mixture of reactive gases for the synthesis: 100 sccm ethylene, 400 sccm or 500 sccm of hydrogen (SmartCem I and SmartCem II respectively) and 600 sccm argon for 120 min. Later all samples were purified from amorphous carbon using a mixture of hydrogen and argon with a flow rate of 100 sccm and 1000 sccm, respectively. After the cleaning process, the samples were cooled down to 200 °C at a rate of 12 °C/min under an inert atmosphere, degassed under vacuum and cooled down to 20 °C in an argon atmosphere.

### 2.2. Materials

An ordinary Portland cement (CEM I 42.5) provided by Cementa-Sweden was used for the synthesis of nanomaterials as well as for the production of all mortar samples. Sieved and cleaned quartz sand having a maximum particle size of 150 µm was used as fine aggregate. The workability of the fresh mixes was controlled by the super plasticizing admixture (sp) type Glenium produced by Grace Chemicals. All mortar mixes had a water-cement ratio (w/c) of 0.35 and a sand-cement ratio (s/c) of 1. Mixes contained: 0 wt% (Ref), 2 wt% (S2), 4 wt% (S4), 6 wt% (S6), 8 wt% (S8), and 10 wt% (S10) of nanomodified cement replacing the untreated cement. The mix proportions are shown in Table 2.

The mortars were mixed using a Bredent vacuum mixer, which minimized the entrapment of air. All samples were cast into Teflon molds without application of any demolding oil and cured in laboratory conditions at 20 ± 2 °C and a relative humidity of 65 ± 5%.

**Figure 2:**
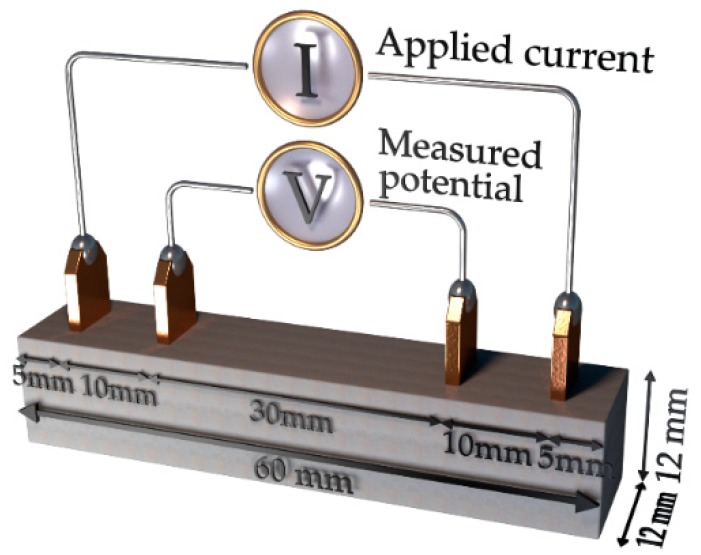
Arrangement of copper electrodes in the mortar sample.

All samples had dimensions of 12 × 12 × 60 mm^3^. Four copper electrodes were made of 0.25 mm thick plates having a width of 5 mm and a height of 15 mm. The copper plates were immersed vertically 7.5 mm deep into the mortar specimen. The distance between the electrodes was 30 mm and between two sets of electrodes 50 mm (Figure 2).

### 2.3. Methods

The nanomodified cement was characterized using a scanning electron microscope (SEM) type Jeol JSM-IT100. All images were obtained with a secondary electron detector (SE). Thermogravimetric (TG) analysis was performed using a thermal analyzer type NETZSCH STA 449 F3 Jupiter^®^ with a temperature increase rate of 10 °C/min and the maximum applied temperature of 1000 °C. 

The electrical resistance was measured using the four-probe method with a digital multimeter type Keysight 34465A. Direct current (DC) was applied between the two outer electrodes and the potential was measured between the two inner electrodes (Figure 2). This configuration showed to have the lowest variation coefficient and a small scatter of the recorded values [35]. The electrical conductivity σ was calculated using the Equation (4):

(4) σ=1/ρ=L/R·A

where: ρ is electrical resistivity, L is the internal electrode distance, A is the electrode area, R = V/I is the measured resistance determined by measuring the voltage drop across the specimen, V is the applied current, I.

**Figure 3:**
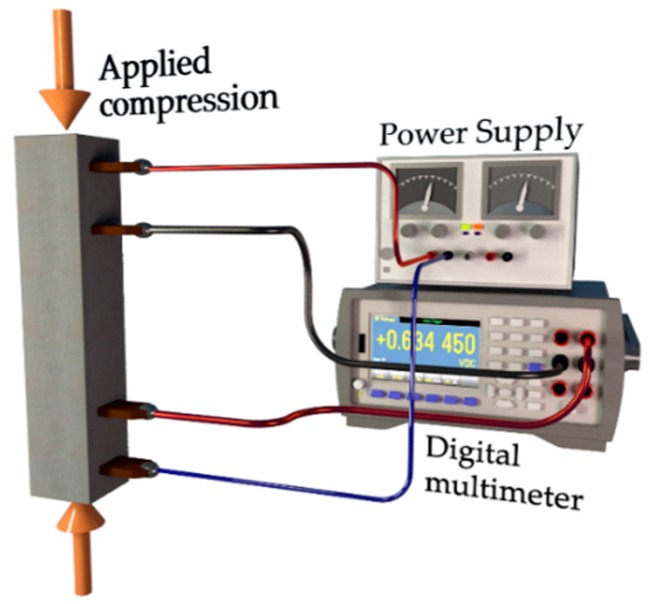
The experimental setup used to measure changes of the electrical resistivity in mortar samples subjected to compression load.

A compression load with a rate of 0.05 cm/min was applied to the vertically placed beam specimens (Figure 3).

**Figure 4:**
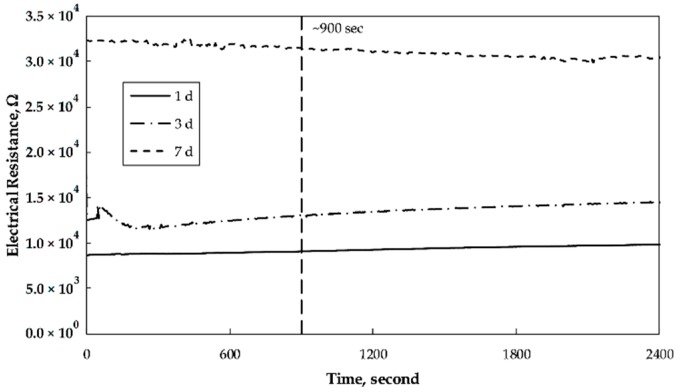
Changes in the electrical resistance with time recorded for mortars containing 8 wt% of the SmartCem I binder 1, 3 and 7 days after casting the specimens.

The presence of moisture in any solidified cementitious matrix causes changes to the electrical conductivity when measured over a longer period of time due to polarization caused by the electrolytic effect. The chemical reactions liberate hydrogen and oxygen, which deposit around the measuring electrodes as a thin film, eventually leading to the polarization effect [36]. Earlier studies confirmed that the measured electrical properties of CNT/cement composites were affected by electrode polarization while using DC [37]. Consequently, to limit that negative effect additional calibration measurements were done to determine the time required to obtain more stable readings before the actual measurement of the electrical resistivity was performed. In the used procedure, the electrical resistivity was measured continuously for 2400 s on mortar samples being 1, 3 and 7 days old. The recorded values are shown in Figure 4. The one-day-old mortars showed a constant value during the entire measuring period which is directly related to a very high moisture content due to a low hydration degree of the Portland cement. The measured electrical resistivity of the 3- and 7-day-old test specimens showed slight variations during the first 600–700 s followed by more stable readings. Based on the repeated measurements it was decided that the values for the determination of the electrical resistance will be recorded with a 900 s delay.

## 3. Results and Discussion

**Figure 5:**
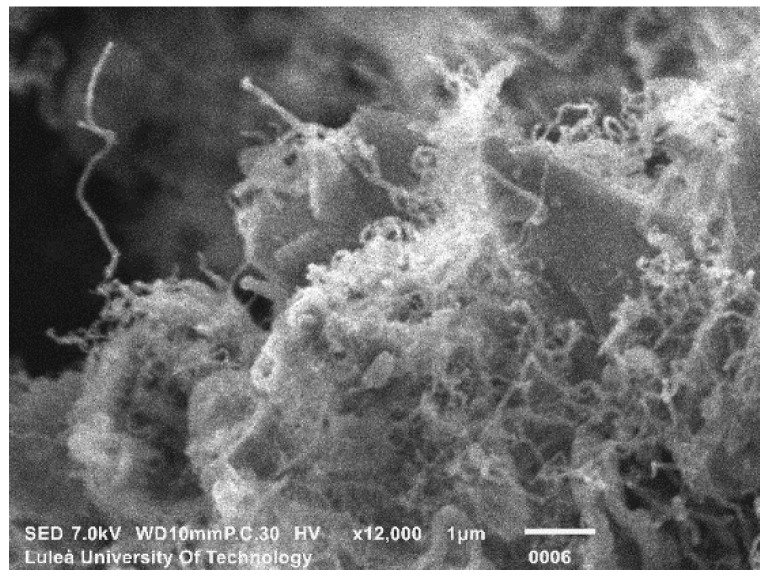
SEM image of SmartCem I.

**Figure 6:**
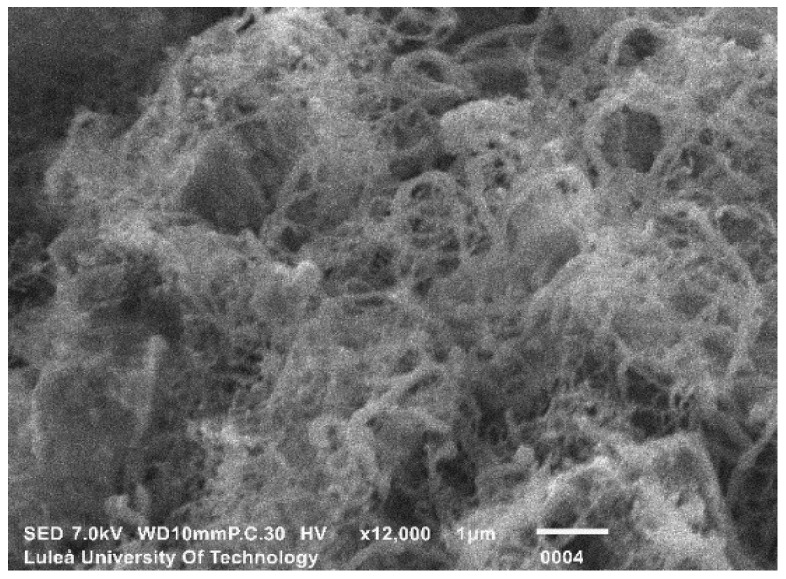
SEM image of SmartCem II.

The SEM images of the nanomodified cements (SmartCem I and SmartCem II) are shown in Figure 5 and Figure 6. All synthesized CNFs were very curly, had diameters between 10–50 nm and lengths between ~3 µm–~20 µm.

**Figure 7:**
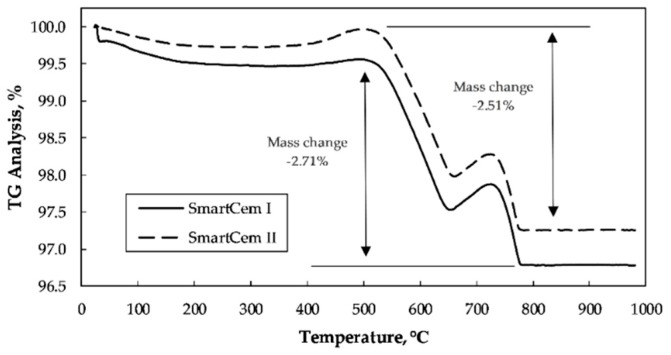
TG Analysis of SmartCem I and SmartCem II.

The nanomodified cements analyzed by TG had two peaks at around 500 °C and 750 °C related to the decomposition of CNFs. The estimated quantities were approximately 2.71 wt% and 2.51 wt% for the SmartCem I and SmartCem II, respectively (Figure 7). The SmartCem I was chosen for further tests due to a lower amount of the hydrogen gas used in the synthesis process and a slightly higher amount of the formed CNFs.

**Figure 8:**
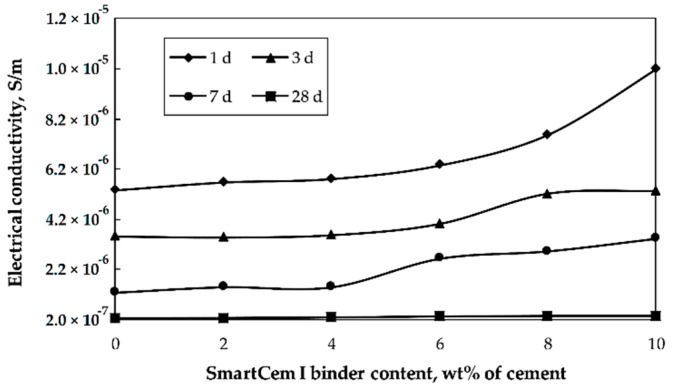
Effects of age and amount of SmartCem I content on the measured electrical conductivity.

The electrical conductivity was determined for the reference mortar and for the composite samples containing SmartCem I binder as replacement of Portland cement between 2–10 wt% of cement (Figure 8).

The electrical conductivity decreased with age for all samples due to the ongoing hydration process consuming the pore water. The pore water provides limited conductivity for the otherwise electrically non-conductive hardened binder matrix. At later ages most of the capillary pore water was consumed thus decreasing significantly the measured electrical conductivity. The most significant change was observed between 7–28 days, which could be related to the densification of the binder matrix with non-conductive phases, including especially calcium silicate hydrate and calcium hydroxide [38]. Replacement of the Portland cement with SmartCem I altered the measured electrical conductivity depending on the age of the sample. At one day the overall conductivity was significantly higher compared to the aged samples; with a maximum value of 5.4 × 10^−6^ S/m. Replacement of 2 and 6 wt% of the untreated cement with SmartCem I slightly increased the conductivity followed by a sharp jump up to 1.0 × 10^−5^ S/m. At higher replacement level, the conductivity of the 7-day-old samples decreased to 1.3 × 10^−6^ S/m and 3.4 × 10^−6^ S/m, respectively. The conductivity of the 28-days-old samples was 2.3 × 10^−7^ S/m for the reference samples and 3.3 × 10^−7^ S/m for samples containing 8 and 10 wt% of the SmartCem I.

**Figure 9:**
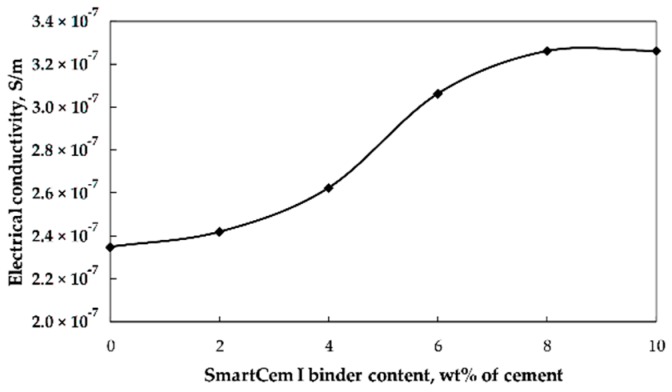
Effects of the SmartCem I content on electrical resistivity of 28 days old samples.

The observed percolation threshold values varied depending on the sample age. In the case of the one-day-old specimen, the maximum conductivity value was reached at the 10 wt% replacement level. In this case, a higher amount of moisture present in the binder matrix created a conducting medium enhancing the measured electrical conductivity. During the following three weeks, the progressing hydration consumed the water and lowered the amount of the conductive medium, which decreased the percolation threshold stepwise from 10, 7 and 5 down to approximately 4 wt% of the SmartCem I. These values corresponded to 0.271, 0.189, 0.135 and 0.108 wt% of CNFs as estimated based on the TG test results. The lowest, 2 wt% of the SmartCem I (corresponding to 0.054 wt% of CNFs), has a minimal effect on the electrical conductivity (Figure 9). The percolation threshold values estimated earlier for Portland cement-based matrixes incorporating multiwall carbon nanotubes oscillated at around 0.20 wt% [39,40]. Whereas values measured in the present research were significantly lower at 0.108 wt% of CNFs. Several factors could contribute to this difference; including CNFs morphology, their distribution within the binder matrix, distance between fibers as well as the microstructure and composition of the isolative binder matrix.

The morphology of the CNFs was shown to affect the percolation threshold and the maximum conductivity. For example, curly shaped CNFs incorporated into polymer-based matrixes increased the percolation threshold concentration from 0.05 to 0.4 vol% [41].

**Figure 10:**
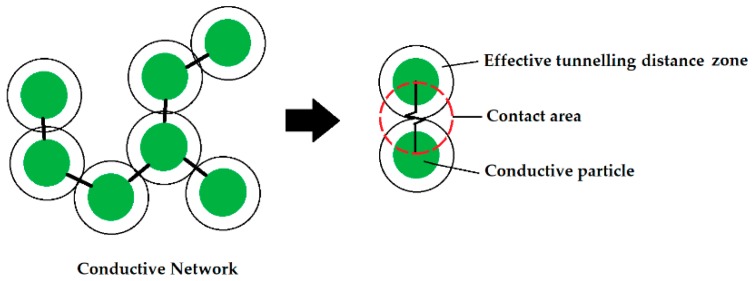
The tunneling effect between conductive particles dispersed in an electrically non-conductive composite.

Furthermore, not all CNFs take part in the conduction despite being evenly distributed within the binder matrix due to being too far apart from each other due to their shape or too low amount. The distance providing conductivity is estimated to be just a few nanometers [42]. The overall electrical conductivity depends also on the number of formed percolated networks. The nanofibers do not touch directly each other due to the Van der Wall forces and the conduction occurs through the tunneling effect [43] (Figure 10).

The tunneling effect is affected by the interfacial transition zone (ITZ) forming between the binder matrix and the CNFs surface. Properties of that zone; its thickness; porosity as well as the chemical composition will strengthen or weaken the effect. The ITZ formed in Portland cement-based matrixes around sand particles and aggregates may range from a few µm to a few hundreds of µm [44,45]. Presumably, in the case of nanosized CNFs the actual ITZ will be only a few nanometers wide. The interfacial transition zones were also observed in polymer-based matrixes incorporating CNTs [46]. Consequently, the observed in the present data decrease of the percolation threshold with age can be related to the general densification of the bulk binder matrix and of the interfacial transition zones.

**Figure 11:**
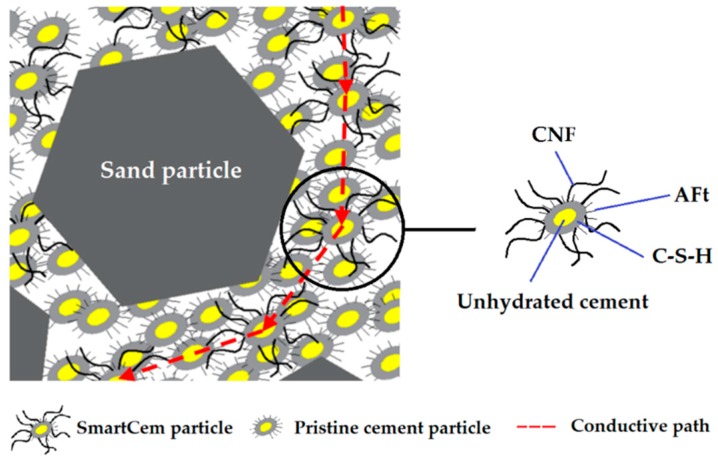
SmartCem with attached carbon nanofibers in hydrated cement matrix.

After 28 days the ultimate measured conductivity of 3.3 × 10^−7^ S/m was constant for the 8 and 10 wt% replacement levels. The CNFs incorporated into a cement matrix are less homogenously dispersed in comparison with polymer matrixes due to the presence of sand particles and anhydrous cement particles. Attaching CNF directly to the cement particles ensures a very high initial dispersion homogeneity and acts as a carrier during the mixing process. It prevents the formation of agglomerates, but at the same time it creates large non-conductive volumes of unhydrated cement particles [45,47] (Figure 11).

**Figure 12:**
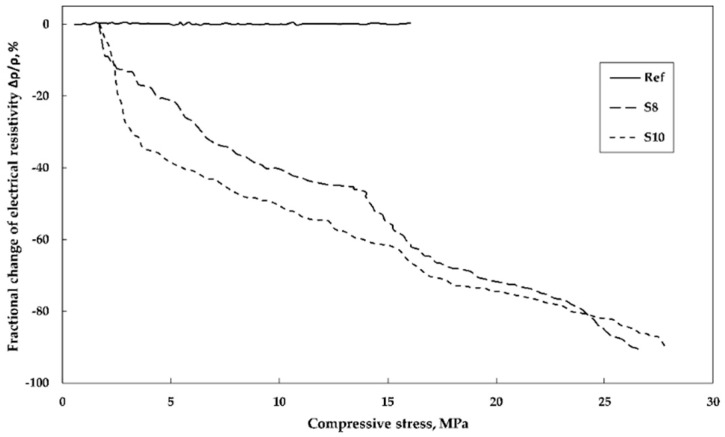
Relationship between fractional change in electrical resistivity and compressive stress.

The piezoresistive response to the applied load was determined on samples produced from three mixes: reference, S8-containing 8 wt% of SmartCem I and S10-containing 10 wt% of the SmartCem I. The recorded dependence between the applied compressive stress and the corresponding measured electrical resistance is shown in Figure 12.

**Figure 13:**
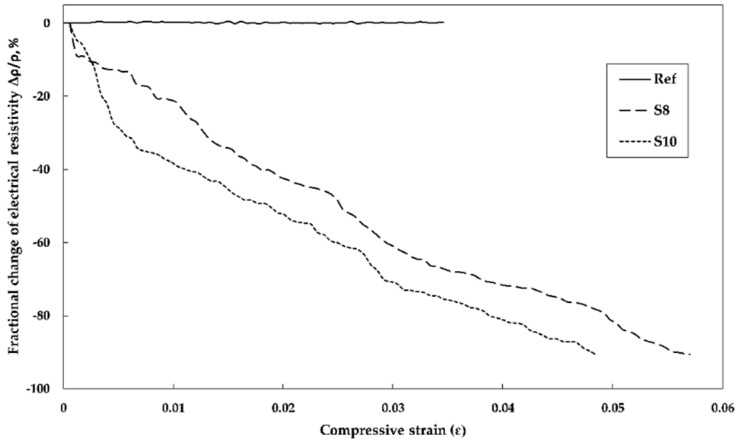
Relationship between fractional change in electrical resistivity and compressive strain.

Both samples, S8 and S10 showed a strong piezoresistive response within the two stages of nearly linear relationships. In the first stage while loading up to 3.5 MPa load, the fractional change of the electrical resistivity reached around 17% and 32% for S8 and S10 respectively. In the second loading stage between 3.5 and 26 MPa the change reached 90%. Portland cement-based mortar incorporating SmartCem I showed also a significant response to strain. The resistivity decreased with increasing compressive strain until the failure occurred (Figure 13). The maximum strain sensitivity of the gauge factor (GF) in this study was calculated based on the fractional resistance change to the change of strain, and equaled to 18.7.

Earlier studies showed a considerably lower sensitivity. For example, Zhang et al. [48] or Yu and Kwon [49] measured between 6 and 9% change in the electrical resistivity for applied loads of 4 MPa and 5.2 MPa. In both cases, the samples were produced by dispersing 0.06; 0.1 wt% and 2.14 vol% of MWCNTs in water (Table 3).

The observed differences can be related, as in the case of the electrical conductivity described earlier, to a number of factors. These include dispersion and morphology of the CNFs/CNTs, their dimensions, curliness but also the microstructure of the binder matrix and the presence of non-conductive inclusions like unhydrated cement particles or sand. In the present case, presumably the strongest effect on the enhanced sensitivity was achieved by the better dispersion of the CNFs. The enhanced dispersion, compared to especially at high pH unstable water dispersions, was obtained by growing CNFs directly on the cement particles. 

The piezoresistive response can be related to the intrinsic piezoresistive property of the CNFs themselves, which was observed in earlier studies on films made of single-wall carbon nanotubes. These results showed a nearly linear relation between the applied strain and the measured voltage and were successfully used for strain sensing [50]. The second phenomenon associated with the piezoresistive response is related to changes of the electrical resistance of the contact points between fibers due to the applied load. In this case, the applied load will presumably compress the binder matrix. CNTs incorporated into a polymer matrix decreased the resistivity at the applied compression load [51,52,53]. A more intensive change of the electrical resistivity in the first stage of the response could be related to the initially stronger densification effect of the more porous part the binder matrix (Figure 12). This could lead to a significant enhancement of the tunneling effect and to the ultimate increase of the electrical conductivity.

## 4. Conclusions

Carbon nanofibers were directly synthesized on Portland cement particles using chemical vapor deposition in the presence of a mixture of ethylene and hydrogen. The produced materials contained 2.71 wt% and 2.51 wt% of CNFs. Percolation thresholds corresponding to the increase of the electrical conductivity were observed in all samples. The threshold tended to decrease with the ongoing hydration of the Portland cement. The percolation threshold varied between 0.271, 0.189, 0.135 and 0.108 wt% CNFs which corresponded to 10, 7, 5 and 4 wt% of the SmartCem I respectively. The studied mortars showed an extremely strong piezoresistive response to the applied compressive load reaching 17% change at 3.5 MPa and 90% at 26 MPa. The piezoresistive response was related to the intrinsic piezoresistive property of the CNFs and to changes of the electrical resistance at the contact points between fibers. Based on the obtained test results from this initial study, the developed material appears to be potentially suitable for applications for stress sensors and smart concrete structures. Furthermore, the measured piezoresistive response should also be sufficient to determine other changes, including, for example humidity, temperature or crack formation. The current ongoing research focuses on those aspects.

## Figures and Tables

**Table 1:** Synthesis parameters.

Named	Argon (sccm)	Ethylene (sccm)	Hydrogen (sccm)	Synthesis Temperature (°C)	Duration (min)
SmartCem I	600	100	400	750	120
SmartCem II	600	100	500	750	120

**Table 2:** Mix proportions used for test mortars.

Mix	w/c	s/c	sp (wt% of Cement)	Cement (kg/m^3^)	SmartCem (wt% of Cement)
Ref	0.35	1.0	0.8	1157	0
S2	0.35	1.0	0.8	1134	2
S4	0.35	1.0	0.8	1111	4
S6	0.35	1.0	0.8	1088	6
S8	0.35	1.0	0.8	1065	8
S10	0.35	1.0	0.8	1042	10

**Table 3:** Load sensitivity of cement/CNT composites measured by others and compared with recalculated results obtained for mix S8.

Publication	Amount of Carbon-Based Materials	(Load MPa) Resistance Change, %	(Load, MPa) Resistance Change, %
Yu & Kwon [49]	0	(5.2)	(8.6)
		0.0	0.0
Yu & Kwon [49]	0.06 wt% MWCNT	(5.2)	(8.6)
		8.8	10.3
Yu & Kwon [49]	0.10 wt% MWCNT	(5.2)	(8.6)
		8.4	11.4
Zhang et al. [48]	2.14 vol% MWCNT	(4)	-
		6.8	
Present result S8	0.20 wt% CNF	(3.5)	(26)
		~17	~90
